# Electrical Properties and Strain Sensing Mechanisms in Hybrid Graphene Nanoplatelet/Carbon Nanotube Nanocomposites

**DOI:** 10.3390/s21165530

**Published:** 2021-08-17

**Authors:** Xoan F. Sánchez-Romate, Alberto Jiménez-Suárez, Mónica Campo, Alejandro Ureña, Silvia G. Prolongo

**Affiliations:** Materials Science and Engineering Area, Escuela Superior de Ciencias Experimentales y Tecnología, Universidad Rey Juan Carlos, Calle Tulipán s/n, Móstoles, 28933 Madrid, Spain; monica.campo@urjc.es (M.C.); alejandro.urena@urjc.es (A.U.); silvia.gonzalez@urjc.es (S.G.P.)

**Keywords:** carbon nanotubes, graphene nanoplatelets, electrical properties, hybrid nanocomposites, strain sensing

## Abstract

Electrical and electromechanical properties of hybrid graphene nanoplatelet (GNP)/carbon nanotube (CNT)-reinforced composites were analyzed under two different sonication conditions. The electrical conductivity increases with increasing nanofiller content, while the optimum sonication time decreases in a low viscosity media. Therefore, for samples with a higher concentration of GNPs, an increase of sonication time of the hybrid GNP/CNT mixture generally leads to an enhancement of the electrical conductivity, up to values of 3 S/m. This means that the optimum sonication process to achieve the best performances is reached in the longest times. Strain sensing tests show a higher prevalence of GNPs at samples with a high GNP/CNT ratio, reaching gauge factors of around 10, with an exponential behavior of electrical resistance with applied strain, whereas samples with lower GNP/CNT ratio have a more linear response owing to a higher prevalence of CNT tunneling transport mechanisms, with gauge factors of around 3–4.

## 1. Introduction

In the last years, the increasing complexity of structures in civil engineering, aerospace, and wind industry has led to the development of new techniques for structural health monitoring (SHM). Many of them are based on conventional non-destructive testing, guided lamb waves, or optical fibers, which have demonstrated a good reliability in detecting damage and failure extent [1,2,3]. However, measurements using these techniques are often difficult as the data acquisition is not easy and most of them only give local information of the structural damage [4,5,6].

In this regard, carbon nanoparticles are now attracting the interest of many researchers as they exhibit outstanding electrical and mechanical properties because they have Young’s moduli of ~270–950 GPa for CNTs [7,8] and ~1 TPa for graphene [9,10] and electrical conductivities of $10^{3}-10^{6}\;S/m$ for CNTs [11,12] and $6000-10^{7}\;S/m$ in the case of graphene nanomaterials [10,13]. Their addition into an insulator resin, in fact, induces the creation of electrical pathways by creating a percolating network [14,15,16,17]. This made possible their use in SHM applications by means of measurements of the electrical resistance variation between two electrodes when the electrical percolation network is modified by strain or cracking [18,19,20,21].

SHM in polymeric composites doped with carbon nanoparticles is based on the fact that the electrical resistivity of nanocomposites changes with applied strain and damage extent. This is owing to the dominant role of the electron tunneling transport [22] as well as the carbon nanoparticle inherent piezoresistive behavior [23,24]. Tunneling resistance increases with applied strain, as it exponentially depends on the distance between adjacent particles [25,26]. Furthermore, the extent of damage induces a disruption of electrical pathways, also leading to an increase in electrical resistance [27]. Therefore, the whole material itself acts as a sensor.

To date, SHM with carbon nanoparticles is focused mainly on CNT and graphene nanoplatelet (GNP)-based nanocomposites. As a general trend, it is observed that GNP-based composites exhibit higher sensitivities than CNT-based ones, with gauge factors (GFs), defined by the change on normalized resistance divided by applied strain, that can range from values of 11 to 40 [28,29,30,31] and 1 to 4 [26,32,33] at low strain levels for GNPs and CNTs, respectively. This can be explained because the 2D distribution of GNPs allows higher interparticle distance between adjacent nanoparticles. However, the percolation threshold, defined as the volume fraction in which the insulator material becomes electrically conductive, is much lower on CNT nanocomposites owing to their effective aspect ratio and large surface area [15,34,35,36], so the selection of GNP or CNT will be determined by the specifications required by the sensor.

Therefore, there is an arising interest in exploring the effect that the combination of both types of nanofillers can have on the final properties of the material [37,38]. In fact, the combination of different architectures with hybrid GNP–CNT fillers has been proven to be an effective way to improve the mechanical properties of the final nanocomposite [39]. Moreover, it can be observed that the addition of CNTs to GNP nanocomposites leads to an enhancement of electrical properties owing to the higher electrical conductivity of CNT networks inside the material [38,40,41]. However, the combination of GNP and CNT nanofillers sometimes does not induce a synergistic effect on electrical properties, as the electrical conductivity values of hybrid nanocomposites are below those obtained for CNT nanocomposites [42,43]. This is explained by the role of nanofiller interactions, which is very dependent on the GNP to CNT proportion and, especially, the dispersion state that relies on manufacturing process. Here, although the electrical conductivity of these materials has been widely explored, the main transport mechanisms that dominate their strain-sensing capabilities remain to be investigated.

For this reason, this study aims to explore the influence of nanofiller interactions and dispersion procedure on the electrical conductivity and strain monitoring capabilities in GNP–CNT hybrid nanocomposites. To achieve this, several proportions of GNP to CNT in an epoxy resin were explored in composites manufactured by different sonication conditions in order to explore the role of possible nanoparticle interactions on electromechanical properties. These samples were subjected to electrical conductivity and strain monitoring tests, allowing to obtain a deeper knowledge of the possible synergistic effects between GNPs and CNTs in these types of materials.

## 2. Materials and Methods

### 2.1. Manufacturing of Nanocomposites

The GNPs used in this study were the *M25*, supplied by *XG Sciences*, with an average lateral size of 25 μm and a thickness up to 6 nm. Multi-wall CNTs (MWCNTs) were *NC3150*, supplied by *Nanocyl*, with an average length of 1 μm and a diameter of 9.5 nm. Epoxy resin was a *DGEBA*-based one, with a commercial name *LY 556* (bisphenol A-(epichlorhydrin); epoxy resin (number average molecular weight <700); CAS no. 25068-38-6) cured with a hardener *XB 3473* (60–100% diethyltoluenediamine CAS no. 68479-98-1; 7–13% 1,2-diaminocyclohexane CAS no. 694-83-7) in a stoichiometric proportion of 100 to 23, respectively.

Several nanocomposites with different proportions of GNPs and CNTs were manufactured under the sonication process. First, GNPs were added to the epoxy matrix in the desired proportion. Then, the mixture was subjected to ultrasonication for 20 or 30 min, depending on the conditions, using a horn sonicator *UP400S* supplied by *Hielscher* at a frequency of 24 kHz, 400 W, and amplitude of 50%. After this step, CNTs were added to the GNP mixture; then, it was subsequently sonicated for another 20 or 30 min depending on the conditions (here, S1 condition refers to sonication for 20 min of GNP mixture and an additional 30 min sonication of GNP+CNT mixture whereas S2 condition refers to sonication for 30 min of GNP mixture and an additional 20 min sonication of GNP+CNT). After sonication stage, the mixture was degassed for 15 min to remove the entrapped air and finally the hardener was added in stoichiometric proportions prior to curing. The cure cycle was set at 140 °C for 8 h.

Table 1 summarizes the different dispersion conditions and the nomenclature used for the study. GNP and CNT contents were selected as they are slightly above the percolation threshold for this type of nanocomposites and, therefore, the maximum sensitivities during strain sensing could be achieved [44].

### 2.2. Characterization of Nanocomposites

DC in-plane electrical conductivity measurements were carried out in specimens with dimensions of 10 × 10 × 1 mm^3^, using four samples for each condition. The electrical resistance, R, was determined as the slope of the linear region of the I–V curve and the electrical resistivity was determined considering the sample geometry by applying the following formula:

(1) $$\rho =R\cdot \frac{L}{A}$$

where ρ is the electrical resistivity, L is the distance between the electrodes, and A is their contact area.

The voltage range was set at 0–20 V in the case of the samples with high electrical conductivity (above 0.1 S/m) and at 0–250 V in the case of the samples with low electrical conductivity. The measurements were performed using a *SMU, Keithley Instrument Inc. mod. 2410.*

Microstructural characterization of fractured surfaces was carried out by means of scanning electron microscopy (SEM), using a *Hitachi 3400* model. The samples were coated by sputtering with a thin layer of gold for an adequate observation.

### 2.3. Strain Sensing Test by Electrical Measurements

**Figure 1:**
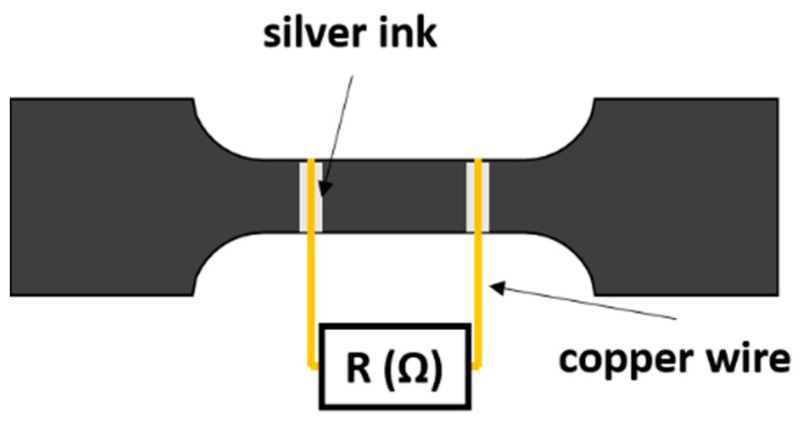
Schematics of electrode’s disposition in strain monitoring tests.

Strain monitoring tests were carried out using three specimens of each conditions to evaluate the SHM capabilities of the proposed materials. Simultaneously, the electrical monitoring was carried out by means of electrical resistance measurements between two electrodes placed in the central region of the bone-shaped samples, as shown in the schematics of Figure 1. These electrodes were made of copper wires sealed with silver ink for a proper attachment to the sample’s surface. The electrical resistance was recorded using an *Agilent 34410 A* module with an acquisition frequency of 10 Hz.

Here, gauge factor was determined as the change of the normalized resistance $\frac{\Delta R}{R_{0}}$ divided by the applied strain, ε.

## 3. Results and Discussion

This section describes the electromechanical behavior of the hybrid GNP–CNT nanocomposites. Firstly, a brief microstructural characterization is carried out. Then, electrical conductivity is deeply explored and, finally, their strain monitoring response is analyzed.

### 3.1. Microstructural Characterization

**Figure 2:**
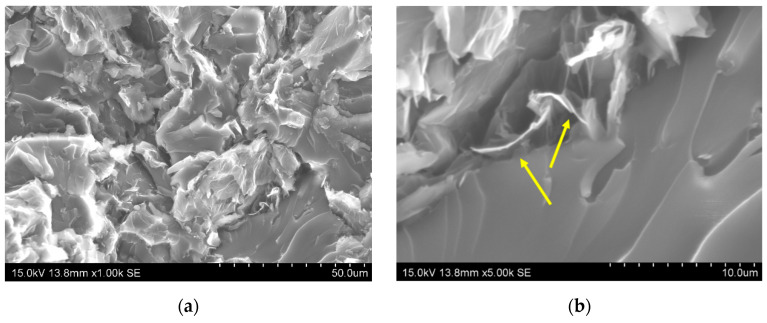

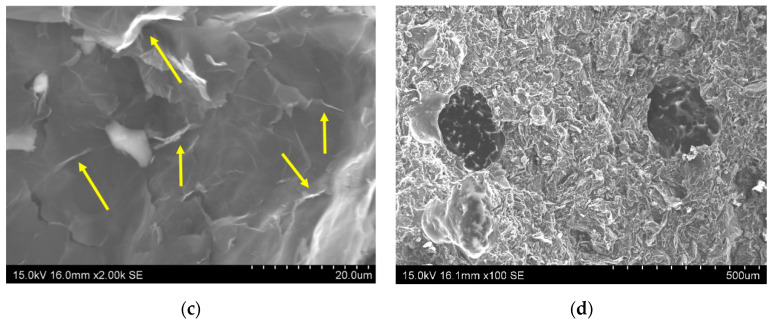
SEM images of the fractured surface of (**a**,**b**) 5GNP-01CNT-S1 and (**c**,**d**) 8GNP-02CNT-S2 conditions (yellow arrows indicate the pull-out of the graphene nanofiller).

Figure 2 shows some SEM images of the fracture of the manufactured hybrid nanocomposites. It can be observed that, in general, the fracture surface is very rough (Figure 2a), which is an indicative of both the crack deviation because its interaction with nanoparticles and of the prevalent pull-out of both the GNPs and CNTs. In fact, this GNP pull-out can be observed at higher magnifications (yellow arrows of Figure 2b). Furthermore, it can be also stated that the GNPs present a much lower lateral size than indicated in the as-received conditions (estimated as 7.3 ± 2.9 μm from the SEM images in comparison with 25 μm of as-received). This is an indicative of prevalent breakage mechanisms during sonication owing to the higher cavitation forces induced [45]. Moreover, it can be also observed that there is a good dispersion of nanofiller inside the epoxy matrix, obtaining a homogeneous distribution, as stated by the yellow arrows of the micrograph of Figure 2c. Furthermore, 8GNP-02CNT samples show an abundant porosity (Figure 2d) that can be associated with a high viscosity of the mixture that prevents adequate removal of the entrapped air during degasification.

### 3.2. Electrical Conductivity Measurements

**Figure 3:**
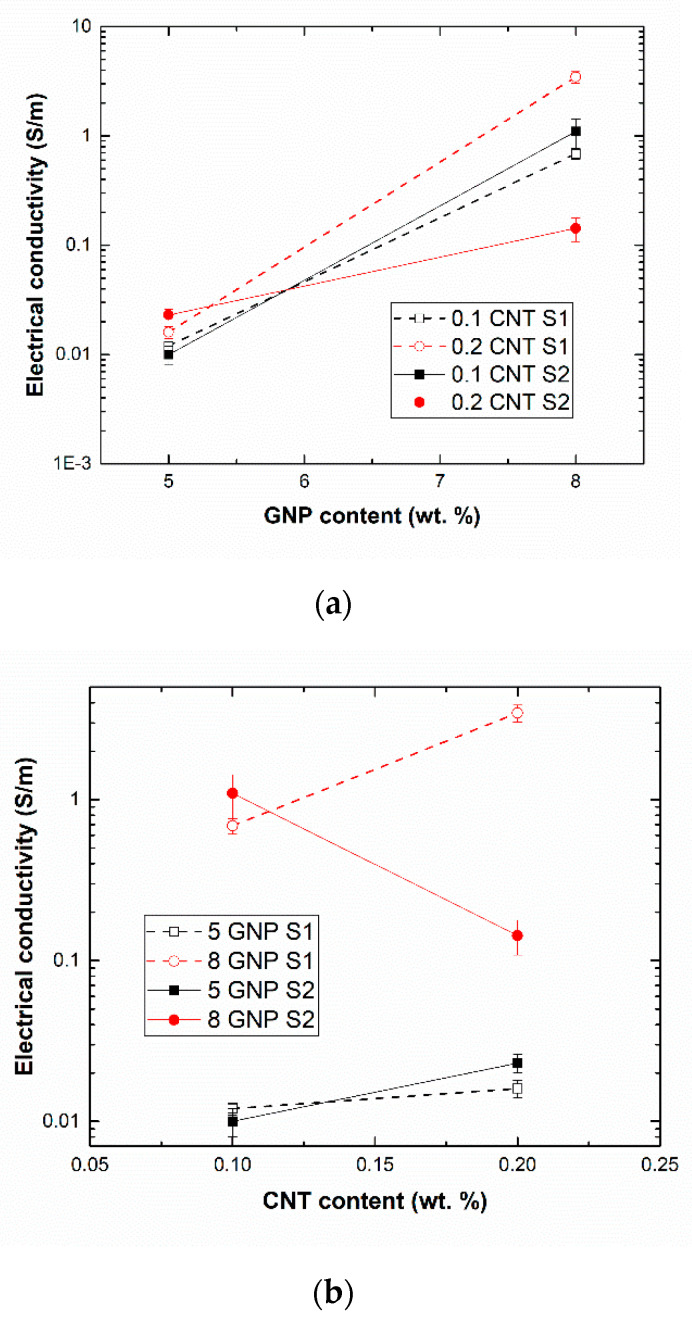
Electrical conductivity values as a function of (**a**) GNP and (**b**) CNT content.

Figure 3 shows the electrical conductivity values measured in the GNP-CNT nanocomposites under different sonication conditions.

Nanofiller disaggregation by the ultrasonication process is achieved by means of cavitation forces, as commented before. These cavitation forces can lead to two different effects: on the one hand, the nanofiller disaggregation as well as a significant exfoliating effect on the GNPs [46] and, on the other hand, a breakage of the nanoparticles as a result of these aggressive cavitation forces. The latter is very dominant in the case of CNTs owing to their high aspect ratio, and thus low bending strength [47,48]. These two mechanisms act in an opposing manners. On the one hand, the exfoliation and disaggregation of nanofillers promote a better dispersed mixture, with lower agglomerates, as well as an increase of the effective aspect ratio of the nanofiller. This leads to a reduction of the percolation threshold [32,35], defined as the critical volume fraction at which the original insulating material becomes electrically conductive. This decrease in percolation threshold induces an increase in the electrical conductivity. On the other hand, however, the breakage of nanofillers due to excessive cavitation forces favors the reduction of the effective aspect ratio of the nanoparticle, causing an increase in the percolation threshold, and thus a decrease in the electrical conductivity.

Therefore, the crucial factor to be determined is the prevalence of each mechanism for the different condition in order to explain the electrical conductivity measurements.

In this regard, it is known that the cavitation forces show a higher influence when the media has a low viscosity [49], meaning that the sonication process is much more effective in this case. Therefore, the optimum sonication time to achieve a well disaggregated network without inducing any prevalent nanofiller breakage must be reduced.

At first sight, it can be noticed that the highest values of electrical conductivity are achieved when increasing the GNP content (Figure 3a). This can be easily explained by the higher amount of conductive nanofiller, which induces the creation of more conductive pathways in the electrical percolating networks inside the nanocomposite. However, the change of the electrical conductivity when analyzing the effect of CNT content is rather more complex, as observed in Figure 3b. Here, two different trends can be observed depending upon sonication conditions; on the one hand, there is an increase of electrical conductivity for both GNP contents at S1 conditions. However, for the S2 conditions, the electrical conductivity increases at 5 wt. % GNP content, but decreases when a 8 wt. % GNP is added. Therefore, it can be concluded that the main electrical transport mechanisms are dominated by the GNP network rather than the CNT one.

**Figure 4:**
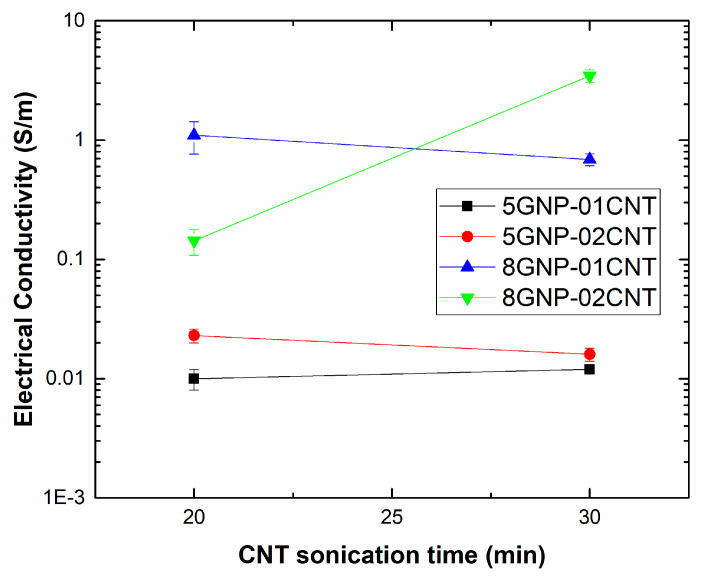
Influence of CNT sonication time on electrical conductivity values for the different tested conditions.

In this regard, it can be noticed that, by adding a content of 5 wt. % GNP, sonication time does not seem to have a very prevalent effect, as the electrical conductivity under S1 and S2 conditions is very similar. However, very important differences can be observed in the case of 8 wt. % GNP samples. Here, it can be noticed that, in the samples containing only 0.1 wt. % CNTs, the electrical conductivity decreases from the S1 to S2 condition, whereas in the case of the samples with 0.2 wt. % CNTs, the opposite trend can be distinguished, appreciating a very significant increase in the electrical conductivity from the S1 to S2 sonication condition. This can be explained by the effectiveness of the sonication process. The total sonication time at which GNPs were subjected was the same under both conditions (50 min), so the differences in the electrical conductivity are attributed to the different sonication times at which the CNT network is exposed. Here, it can be observed that increasing the sonication time of CNT fraction (Figure 4) leads to a decrease in conductivity of 8 GNP-0.1 CNT samples and an increase in the case of 8 GNP-0.2 CNT ones. When increasing the CNT content, the viscosity of the media also increases, owing to the 1D nature of the nanofillers that leads to a significant increase even at such low contents [50,51]. Therefore, the effectiveness of the sonication process is lower and, because of that, the optimum sonication time increases. At lower CNT contents, the viscosity of the media is lower, so the sonication effectiveness is higher and the optimum conductivity values are achieved at lower sonication times. Under this condition, when a longer sonication time is applied (30 min), the CNT breakage effect is more prevalent, leading to a reduction of the electrical conductivity, as observed in the S1 condition.

Furthermore, when comparing the electrical conductivity measured in the 8GNP-02CNT nanocomposite at S1 conditions with other studies dealing with only GNPs or CNTs, a significant increase of this property is observed: from 10^−3^ S/m for 8 wt. % GNP nanocomposites [52] and 0.1 S/m for 0.3 wt. % CNT nanocomposites [32] to 3 S/m reached in this work. Therefore, a synergistic effect is observed under these conditions, indicating that the distribution of both GNPs and CNTs makes possible the creation of a more efficient electrical network.

### 3.3. Strain Monitoring Tests

**Figure 5:**
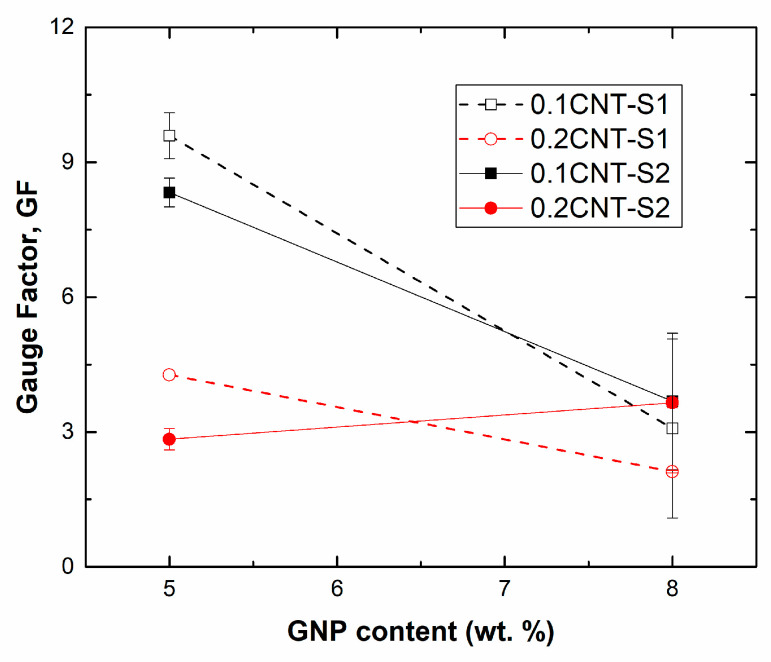
Values of gauge factor at a strain level of ε=0.005 for the different tested conditions.

Figure 5 summarizes the values of the gauge factor, calculated as the variation of the normalized resistance divided by the applied strain at low strain levels (ε=0.005), as a function of the GNP content. Here, it can be noticed that the sensitivity is mainly dominated by the content and the type of the nanofiller rather than the dispersion procedure.

In this regard, it can be observed that, for the samples with 0.1 wt. % of CNTs, the electrical sensitivity drastically decreases with the increasing GNP concentration from 5 to 8 wt. %. Therefore, it can be stated that, at these proportions of GNP to CNT, the GNPs seem to dominate the formation and stability of the electromechanical network. However, when increasing the amount of CNTs to 0.2 wt. %, no significant differences are observed in GNP content. Here, the proportion of CNTs to GNPs is higher, thus contributing in a more prevalent way to the changes in the electrical behavior with applied strain.

Concerning the dispersion procedure, as commented before, no prevalent changes were observed for the different sonication times applied. At lower GNP contents, it is seen that increasing the sonication time of CNTs from 20 to 30 min (S2 to S1 condition) induces a slight increase of the gauge factor. This can be explained by the previously explained breakage effect of the CNT by the cavitation forces, which leads to a reduction in the effective aspect ratio of the nanotubes, and thus to an increase in the percolation threshold [35,53]. A higher percolation threshold promotes an increase in the interparticle distance, leading to a more prevalent tunneling effect [22,25], and thus to a higher sensitivity, according to J. G. Simmons’ formula to estimate the electrical transport due to tunneling effect [54]:

(2) $$R_{tunnel}=\frac{h^{2}t}{Ae^{2}\sqrt{2m\varphi }}exp\left(\frac{4\pi t}{h}\sqrt{2m\Phi }\right)$$

where $R_{tunnel}$ is the tunneling resistance; *t* is the interparticle distance or tunneling distance between adjacent nanoparticles; *h* is Planck’s constant; *m* and *e* are the electron mass and charge, respectively; *A* is the area in which electrical transport takes places or tunneling area; and *φ* is the height barrier of the matrix.

At higher GNP contents, the opposite effect with sonication time is observed, with a decrease in the gauge factor when increasing the sonication time from S2 to S1 conditions. Here, as explained before, the cavitation forces are not so effective owing to the higher viscosity of the mixture, so there is no prevalent breakage of the nanoparticle, and thus the reduction of the effective aspect ratio is not remarkable.

The values of the GF for the best conditions (5GNP-01CNT) are considerably higher than the sensitivities reported in other studies [32], where GF values were reported to be in the range of 2–4. Moreover, these values are also significantly higher than conventional metallic gauges, with GFs typically around 2 [55].

**Figure 6:**
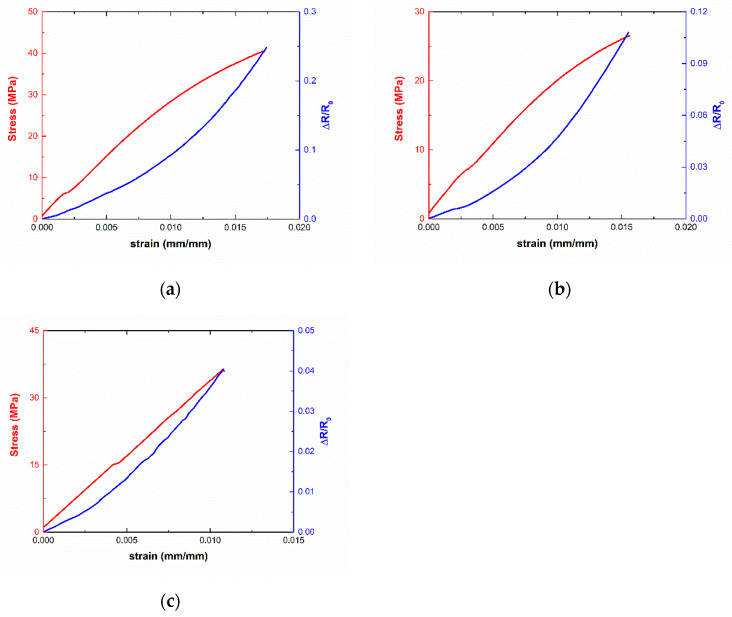
Electromechanical curves of (**a**) 5GNP-01CNT-S1, (**b**) 8GNP-02CNT-S1, and (**c**) 5GNP-02CNT-S1 samples.

**Figure 7:**
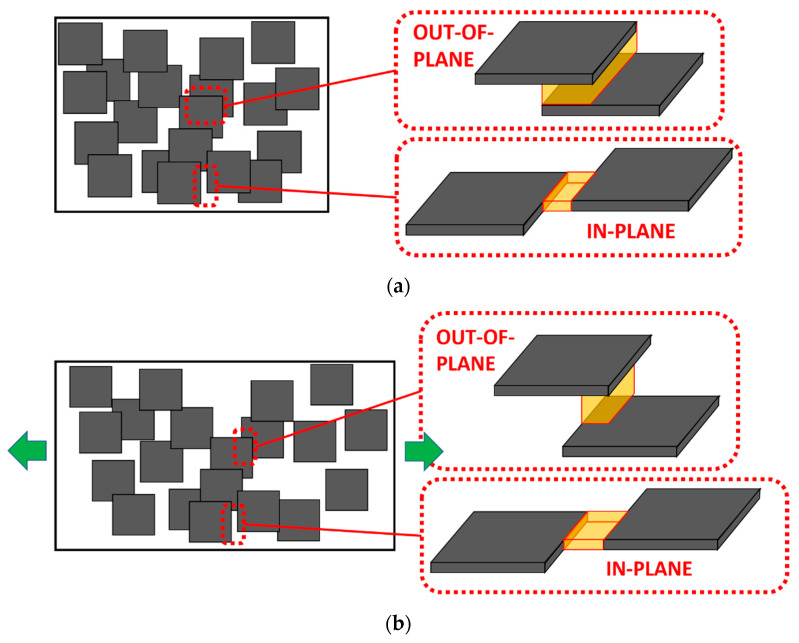

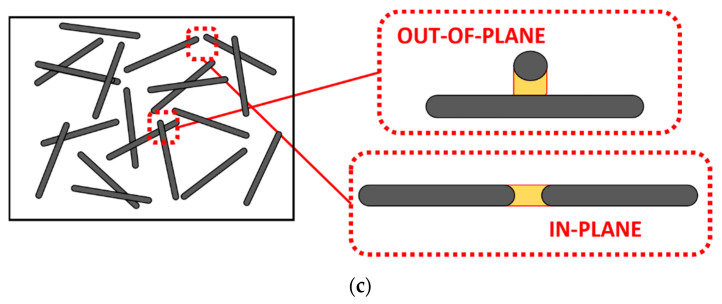
Schematics of out-of-plane and in-plane contacts between adjacent nanoparticles for (**a**) GNPs at initial state and (**b**) after strain is applied and (**c**) CNTs.

Furthermore, Figure 6 shows three examples of the electrical resistance variation with applied strain. Here, it can be observed that there is a marked exponential correlation between the changes in the electrical resistance and the applied strain. This behavior has been explained in previous studies and it is more typical of GNP-based nanocomposites [30,56]. It can be associated with the ways of interacting neighboring particles inside the nanocomposite. They can be mainly in the form of both in-plane and out-of-plane contacts [57], as observed in the schematics of Figure 7. When observing these interactions among GNPs, it can be stated that the tunneling area between adjacent nanoplatelets is significantly different from out-of-plane to in-plane contacts (Figure 7a). Therefore, the tunneling resistance associated with out-of-plane and in-plane contacts changes in a very different way.

In this context, at low strain levels, the tunneling resistance variation is mainly dominated by out-of-plane contacts [30], which explains the lower gauge factor, as the out-of-plane tunneling resistance is dominated by the Poisson effect. On the other hand, at high strain levels, there is a predominance of in-plane contacts as the tunneling area due to out-of-plane contacts is reduced, as shown in the schematics of Figure 7b, which explains the higher variation of electrical resistance with applied strain.

However, in the case of CNTs, there is no significant different between the tunneling areas of out-of-plane and in-plane contacts (Figure 7c), so the variations of the electrical resistance with applied strain follow a much more linear behavior, as observed in previous studies [26,32].

**Figure 8:**
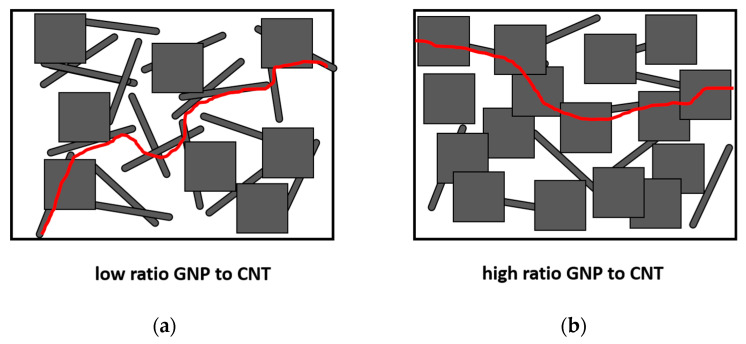
Preferential conductive pathways (highlighted in red) in hybrid nanocomposites with: (**a**) low ratio GNPs to CNTs and (**b**) high ratio of GNPs to CNTs.

In this regard, when comparing samples with a higher proportion of CNTs, such as 5GNP-0.2CNT, a more linear trend between the variation of electrical resistance and applied strain is observed (Figure 6c). In these specimens, CNTs play a highly dominant role in the formation of the electrical percolation network of the nanocomposite in comparison with the samples with a higher GNP/CNT ratio, which show similar gauge factors to GNP nanocomposites [30], as the prevalent electrical pathways are dominated by the role of GNPs, as shown in the schematics of Figure 8. Therefore, the electromechanical curves provide important information about the main mechanisms and interactions that take place in these hybrid nanocomposites as well as the possible synergetic effects that can be achieved.

## 4. Conclusions

Electromechanical properties of GNP/MWCNT-reinforced epoxy matrix nanocomposites were evaluated using different sonication conditions for the nanoparticle dispersion in the polymer.

It was observed that the electrical conductivity increases significantly with GNP content owing to their prevalence in the creation of conducting networks inside the nanocomposite. The sonication time has different effects depending on the content of each nanofiller. At low GNP contents, there are no significant variations when increasing the sonication time of CNTs. This is explained by the low viscosity of the media that makes the sonication process more effective at low sonication times. However, at higher GNP contents, the differences among the different sonication conditions are more prevalent. Here, the highest electrical conductivities are achieved at higher CNT contents (0.2 wt. %) with larger sonication times, owing to the higher viscosity of the media that makes the sonication process more effective when the sonication stage is extended.

Strain monitoring capabilities were evaluated by means of electrical resistance measurements during tensile tests. The highest sensitivities were achieved at low GNP/CNT contents owing to the higher prevalence of tunneling mechanisms at contents around percolation threshold. When analyzing in detail the electromechanical curves, it has been observed that, at lower strain levels, there is a prevalence of out-of-plane contacts, whereas at higher strain levels, in-plane mechanisms are the ones that dominate the electrical conductivity variation. For nanocomposites with a high GNP/CNT ratio, there is a more pronounced exponential behavior owing to the higher variation of electrical resistance inside the GNP network with applied strain, as out-of-plane and in-plane contact areas are quite different. However, for the samples with a higher proportion of CNTs, the electrical response with applied strain is much more linear owing to the higher prevalence of CNTs in their electromechanical response.

Furthermore, the high electrical conductivities achieved along with the good sensitivity make these materials very suitable for SHM and electrical applications.

However, it would be necessary to further explore other sonication parameters (i.e., amplitude or energy) to gain a deeper knowledge of the effect of the dispersion procedure in the electrical network of the final nanocomposites. Alternatively, future work should be addressed to develop an analytical model that could properly explain the interactions between GNPs and CNTs based on the hypothesis presented in this study.

## Figures and Tables

**Table 1:** Nomenclature used for materials manufactured and tested.

GNP Content (wt. %)	CNT Content (wt. %)	Sonication Time (GNP—GNP + CNT)	Designation
5	0.1	20–30 min	5GNP-01CNT-S1
	0.2	20–30 min	5GNP-02CNT-S1
	0.1	30–20 min	5GNP-01CNT-S2
	0.2	30–20 min	5GNP-02CNT-S2
8	0.1	20–30 min	8GNP-01CNT-S1
	0.2	20–30 min	8GNP-02CNT-S1
	0.1	30–20 min	8GNP-01CNT-S2
	0.2	30–20 min	8GNP-02CNT-S2

## Data Availability

The data presented in this study are available on request from the corresponding author. The data are not publicly available owing to technical issues.
